# Closed Stereotactic Needle Biopsy of Brainstem Small Cell Glioblastoma: A Case Report and Literature Review

**DOI:** 10.7759/cureus.2559

**Published:** 2018-05-01

**Authors:** Shaheen Jadidi, Anthony D'Abarno, Egon M Doppenberg

**Affiliations:** 1 Cognitive Neurology and Alzheimer’s Disease Center, Northwestern University, Feinberg School of Medicine; 2 Chicago College of Osteopathic Medicine, Midwestern University; 3 Neurological Surgery, Advocate Good Samaritan Hospital

**Keywords:** neurosurgical education, neurosurgery, case report, glioblastoma, brainstem, brain biopsy, glioma, neuro-oncology, neuro-navigation, neuropathology

## Abstract

Glioblastoma is the deadliest and most common of the malignant primary brain tumors that can occur in adults. In contrast, brainstem gliomas are extremely uncommon in adults; however, their precise incidence is not known, due to the difficult nature of obtaining tissue from the brainstem provoking low biopsy and resection rates. In this case report, we have examined a 34-year-old female who was diagnosed with pontomedullary small cell glioblastoma following a successful biopsy of the brainstem lesion. A closed stereotactic needle biopsy with a contralateral approach was utilized using stealth computed tomography (CT) neuronavigation for tissue diagnosis without causing additional neurological deficits. Our goal is to share this novel approach of obtaining tissue from the brainstem in order to aid others in definitively diagnosing brainstem gliomas and subsequently providing appropriate treatment early in the disease process.

## Introduction

Brainstem gliomas represent less than 2% of adult gliomas and consist of a number of anatomical and histological variants that require individualized consideration [1-2]. While adult brainstem gliomas have a median survival of 30 to 40 months, the median survival drops to 14.8 months in cases of brainstem glioblastoma [3]. Small cell glioblastoma is an aggressive variant that clinically manifests like primary glioblastoma [4]. Medullary gliomas fare only slightly better than pontine gliomas, with a median survival of 51.3 months and 25.3 months, respectively [5]. These poor outcomes highlight our current rudimentary understanding of brainstem gliomas, and specifically, small cell glioblastoma. Unfortunately, research on the unique characteristics of brainstem glioma variants is largely limited by the lack of tissue diagnosis, as a biopsy of these lesions is generally forgone due to its significant risk. Here, we report the clinical history and outcomes of a patient with a small cell glioblastoma of the brainstem who underwent a novel stereotactic needle biopsy, which allowed for accurate diagnosis and directed treatment.

## Case presentation

A 34-year-old Caucasian female presented to the emergency department with complaints of right hemiparesis, numbness, dysphagia, and ataxia. These complaints began as numbness over her right scapular area two weeks prior, and the numbness later progressed to include the right leg. Additionally, she reported five days of waking up during the night with severe headaches. Although she reported a history of migraines, which were usually accompanied by an aura, she stated that these new headaches were different in nature. She also had no known allergies and no previous surgeries. Her mother had a history of breast cancer, and her father had a history of ischemic heart disease. A review of systems was otherwise negative. After workup in the emergency department and consultation by neurology, she was referred to neurosurgery for management of a high-grade brainstem tumor.

Examination

On initial presentation, the patient was alert and oriented to person, place, and time with a Glasgow Coma Scale (GCS) score of 15. Her pupils were equal, round, and reactive to light. Cranial nerves II-XII were grossly intact. Motor testing revealed that strength was 5/5 in both upper and lower extremities. Pronator drift was noted in the right upper extremity. Dysmetria was noted in the right upper extremity during finger-to-nose testing, and discrimination of fine touch was subjectively diminished on the entire right side from the zygomatic process to the foot. Patellar reflexes were 3+ bilaterally. Over the course of her initial admission, the patient demonstrated a progressive decline, to include mild left facial droop, dysarthria, and a worsening dysphagia. A percutaneous endoscopic gastrostomy (PEG) tube was placed because the patient was unable to swallow without effort. These findings continued until her initial discharge. Upon readmission, the patient displayed similar findings. Left lower quadrant abdominal pain radiating to the shoulder and acute numbness of the left chest and shoulder were also noted. These findings progressed and worsened until the patient was intubated after deteriorating to a GCS score of 10.

Imaging

**Figure 1:**
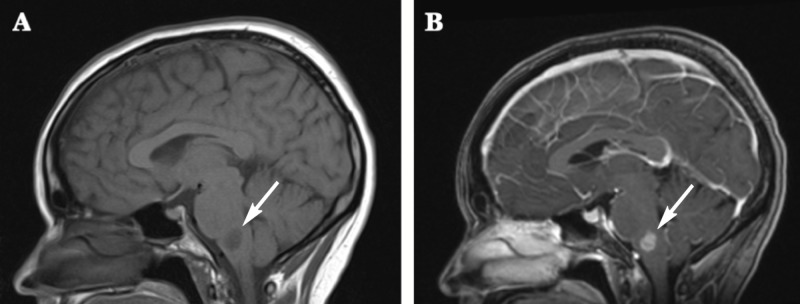
Magnetic resonance imaging (MRI) prior to biopsy. The lesion within the medulla (arrows) demonstrated restricted diffusion and smooth, moderately thick peripheral enhancement with central necrosis (A: T1-weighted image, B: T2-weighted image).

**Figure 2:**
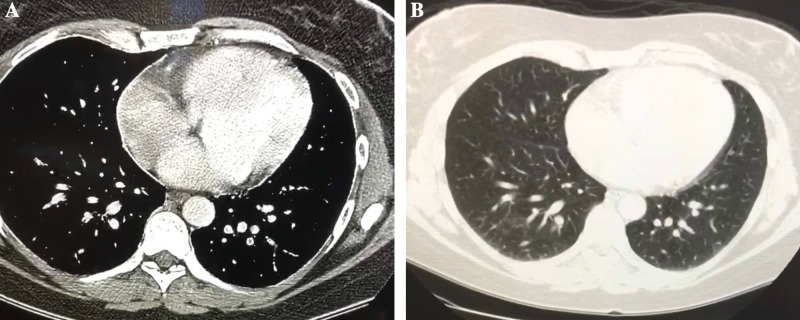
Computed tomography (CT) scans. A: Chest CT with contrast enhancement in the transverse plane, B: Chest CT without contrast enhancement in the transverse plane.

Magnetic resonance imaging (MRI) of the brain with and without contrast revealed a heterogeneous T1 hypointense (Figure 1A) and T2 hyperintense (Figure 1B) signal abnormality involving the pontine base and extending into the medulla. There was mildly increased relative cerebral blood flow and blood volume within the enhancing portion of the abnormality, suggesting neovascularity. The pontine component of the lesion also showed no abnormal enhancement or significant hyperperfusion. Mild mass effect on the fourth ventricle was noted. There was no peritumoral edema or significant mass effect. The remainder of the brain revealed no abnormal enhancement. No abnormal leptomeningeal enhancement was observed. A magnetic resonance imaging (MRI) scan of the spinal column with and without contrast also demonstrated no evidence for metastasis to the cervical, thoracic, or lumbar spine, with normal caliber and signal intensity of the spinal cord. A computed tomography (CT) scan of the chest with (Figure 2A) and without (Figure 2B) contrast enhancement revealed scattered small ground glass and nodular opacities bilaterally. These were nonspecific, with differentials including inflammatory and infectious etiologies, although metastatic foci remained difficult to completely exclude in the context of malignancy. A CT of the abdomen and pelvis with contrast revealed no intra-abdominal mass or evidence of metastatic disease. 

Differential diagnosis

- High-grade glioma

- Primary central nervous system (CNS) lymphoma

- High-grade medulloblastoma

Management

The patient was initially started on dexamethasone 4 mg orally every six hours until imaging was reviewed. Dexamethasone was discontinued due to a concern for possible lymphoma, but shortly thereafter the patient began to complain of worsening ataxia, dysarthria, and dysphagia. Dexamethasone was subsequently resumed.

Operation

**Figure 3:**
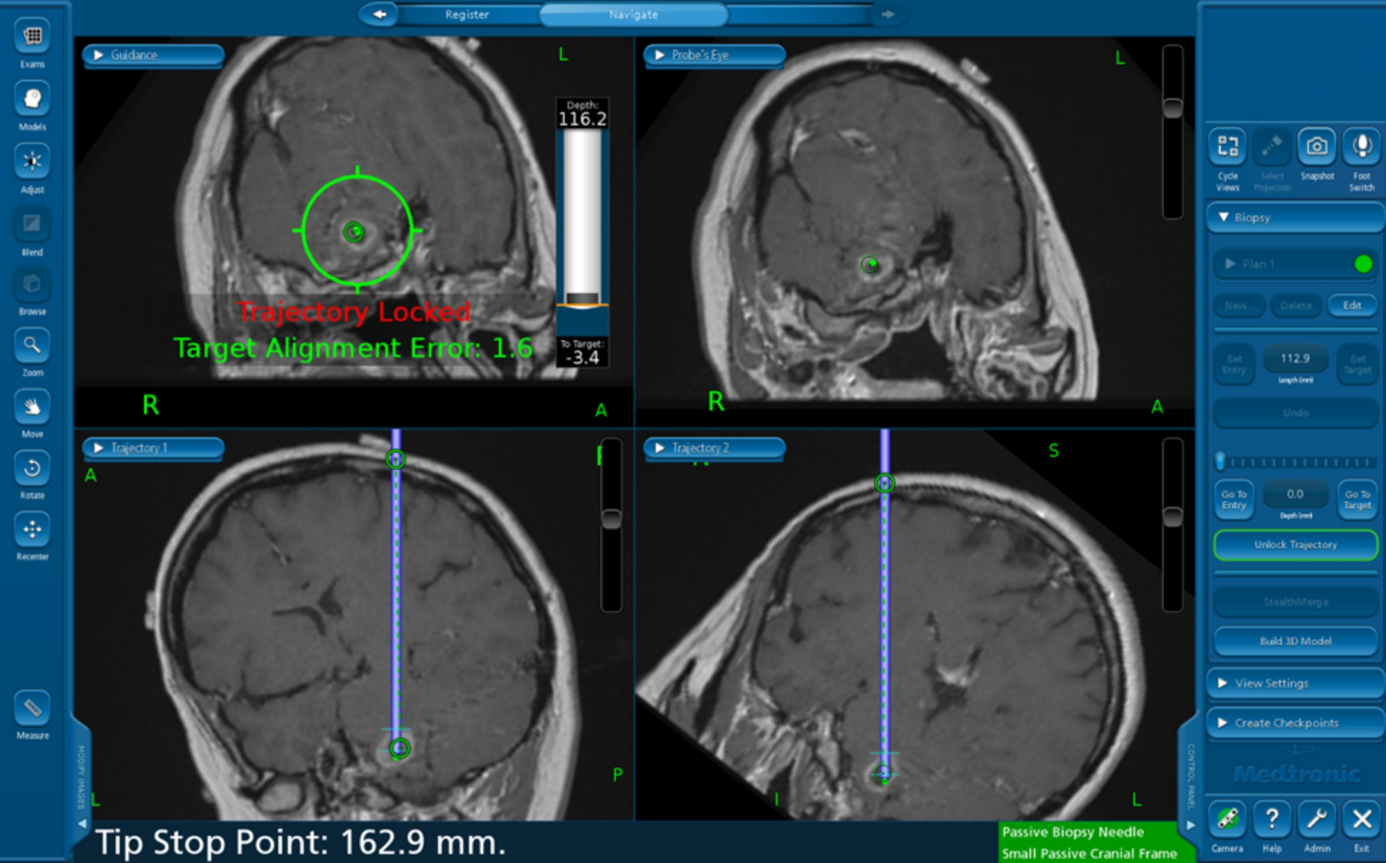
Live image of stealth CT neuronavigation interface during biopsy. Stealth CT neuronavigation allowed surgeons to visualize the anatomy of the patient’s brain during the closed stereotactic needle biopsy. The precise tracking of the location of surgical instruments in relation to the patient’s anatomy and accommodation for brain shift allowed for more precise navigation during surgery. CT - computed tomography.

The patient was transported to the operating room on Day 5 of her hospitalization to undergo a closed stereotactic needle biopsy. Once the patient was anesthesized, the head was secured and the fiducials on the scalp were registered by cameras into the computerized stealth CT neuronavigation system in the operating room. A minimal amount of hair was shaved from the scalp and a small incision was marked out. This area was then meticulously cleaned and draped in a sterile fashion. An opening in the skull about the size of a quarter was made (burr hole), exposing the dura which was then opened. A stereotactic biopsy needle was then introduced with intraoperative use of the neuronavigation system in order to guide the needle to the target using a contralateral approach (Figure 3). Biopsy samples were successfully obtained for pathologic examination. After the incision was closed, a clean and dry dressing was applied. The patient was then extubated in the operating room and transported to the post-anesthesia care unit in stable condition.

Postoperative course

Postoperatively, a nasogastric tube was placed to provide nutrition, due to worsening dysphagia. On Day 10 of her hospitalization, a PEG tube was placed. The patient was scheduled for chemotherapy and craniospinal radiation therapy, and was discharged on Day 12. Four days later, the patient returned due to worsening symptoms, and dexamethasone was increased to 10 mg orally every six hours. The patient was started on temozolomide IV (intravenous) on Day 10 of readmission, but she developed respiratory acidosis/hypercapnia with a CO2 of 115 and was started on a bilevel positive airway pressure (BIPAP), two days later. As a result, a rapid response team was called to transfer the patient to the intensive care unit (ICU). The temozolomide was converted to oral dosing via a PEG tube. The patient’s family was consulted regarding the patient's status and their options. The patient chose to update her status to do not intubate. The patient was extubated prior to her discharge to a hospice care facility, where she passed away four days later.

Pathologic findings

**Figure 4:**
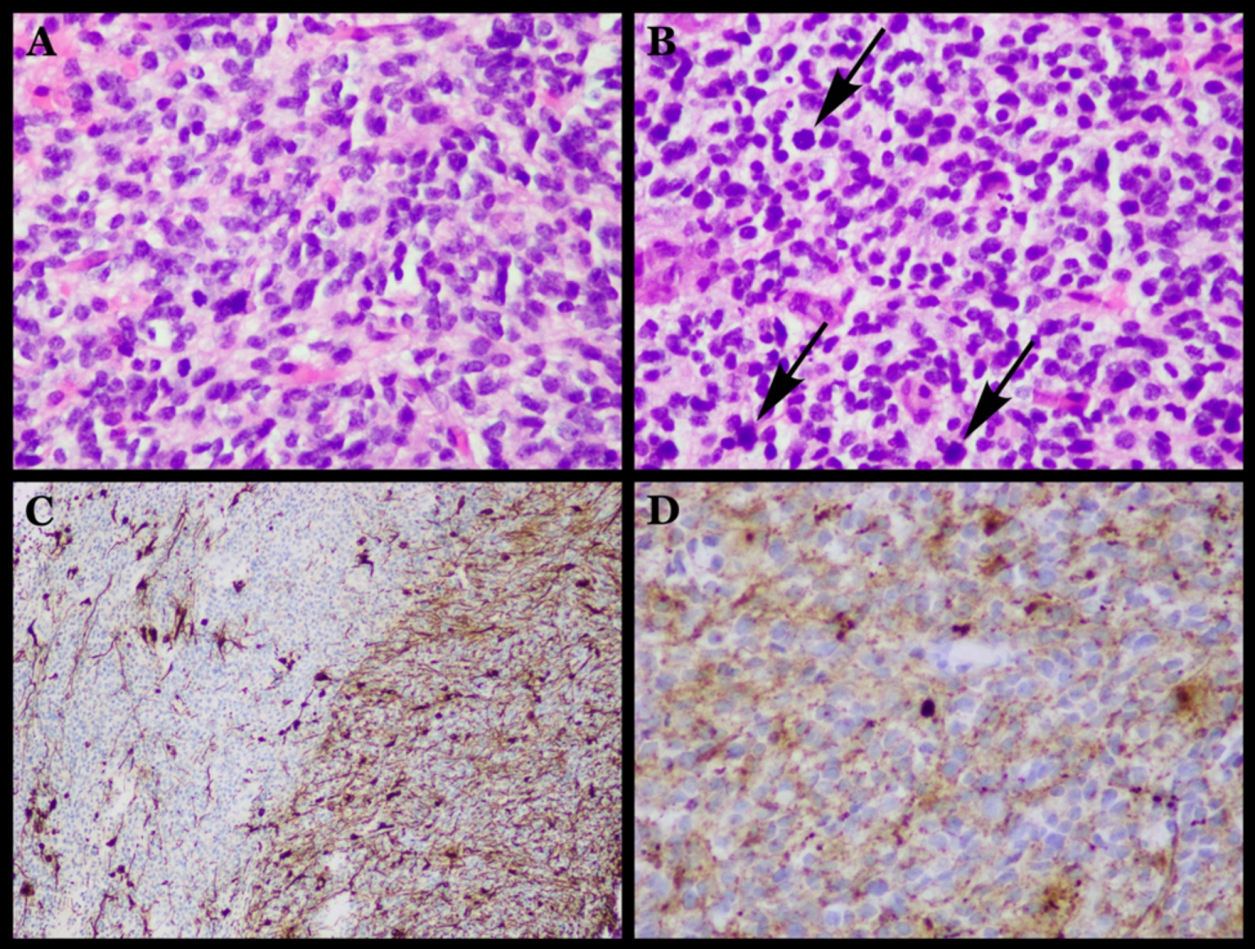
Histological results of pathology studies. The biopsy revealed marked hypercellularity of small round undifferentiated cells with marked pleomorphism and prominent vascularity (A, B). Note the presence of mitotic figures (B; arrows). Immunohistochemical markers such as synaptophysin and glial fibrillary acidic protein (GFAP) are generally interpreted as evidence of differentiation along the neuronal or glial lineage. The presence of brown staining is indicative for GFAP (C), a protein that makes up the intermediate cytoplasmic filaments of normal and neoplastic astrocytes. Synaptophysin is a reliable marker for the identification of normal neuroendocrine cells and neuroendocrine neoplasm, but is not usually expressed in pure glial tumors [6]. The presence of brown staining is indicative of synaptophysin (D).

The brain biopsy was originally interpreted as a classic medulloblastoma (WHO Grade 4). The tumor was composed of sheets of small blue cells with rare poorly formed rosettes (Figure 4A-4B). An immunohistochemical stain for glial fibrillary acidic protein (GFAP) and synaptophysin revealed moderate astrocytosis (Figure 4C) and little synapse loss (Figure 4D), respectively. Reticulin staining was also negative. Staining for p53 was positive, and Ki-67 index was about 50%. The sample was then sent to St. Jude’s Children’s Research Hospital in Nashville, TN, for further molecular analysis. Although their differential diagnosis also included medulloblastoma, the diagnosis was amended at St. Jude’s Children’s Research Hospital to small cell glioblastoma (WHO Grade 4). Their analysis indicated only focal immunoreactivity for GFAP and weak immunoreactivity for synaptophysin. However, immunoreactivity for Olig-2 and p53 was present in a majority of the tumor cells. Interphase fluorescence in situ hybridization  (iFISH) analysis revealed platelet-derived growth factor receptor A (PDGFRA) amplification. No amplification of MYC, NMYC, or epidermal growth factor receptor (EGFR) was observed.

## Discussion

Brainstem gliomas generally represent a poor overall prognosis, as surgical resection is rarely an option. Concurrent radiation and chemotherapy are the mainstays of treatment, but they rarely produce a dramatic effect on the growth of the tumor and have failed to yield desirable outcomes for patients. Immunotherapy approaches such as immune checkpoint inhibitors [7], modified T-cells [8], and signal transducer and activator of transcription 3 (STAT3) inhibitors [9] are currently being investigated. Unfortunately, low mutational load and tumor heterogeneity are hallmarks of glioblastoma, which shortens the list of potential targets for immunotherapy. Even so, the development and implementation of high-throughput technologies have made personalized immunotherapy feasible [10], although this would require successful tissue biopsy, such as the one presented in this case, as a source for tumor-derived information.

## Conclusions

In this case study, the biopsy of a pontomedullary brainstem lesion was successfully obtained by mapping a contralateral trajectory through the patient’s brain using stealth CT neuronavigation. This approach allowed for the avoidance of vital anatomy and vasculature such as the ventricular system, subarachnoid cisterns, and the circle of Willis, without causing any additional neurological deficits. We emphasise the essential need for tissue biopsies in brainstem gliomas without which definitive diagnosis and adequate treatment would be difficult. This is especially important with immunotherapeutic strategies which have already revolutionized the treatment of different malignancies, as tissue biopsies can be analyzed at the cellular, deoxyribonucleic acid (DNA), ribonucleic acid (RNA), epigenetic, protein, and metabolome levels for potential immunotherapeutic targets in brainstem gliomas.
